# Editorial: More than just skin-deep: considering ethnic, racial, and healthcare disparity-based factors in pain experience, treatment, and guidelines

**DOI:** 10.3389/fpsyt.2026.1788988

**Published:** 2026-02-25

**Authors:** Mark Vorensky, Zina Trost, Maisa S. Ziadni

**Affiliations:** 1 Department of Rehabilitation and Movement Sciences, Rutgers University School of Health Professions, Newark, NJ, United States; 2 Department of Psychological and Brain Sciences, Texas A&M University, College Station, TX, United States; 3 Department of Anesthesiology, Perioperative and Pain Medicine, Stanford University School of Medicine, Palo Alto, CA, United States

**Keywords:** decolonizing knowledge, disparities, equity, inequities, life course, pain, pain measurement, racism

## Introduction

This Research Topic features four articles with distinct social contexts that converge on a shared imperative: to understand and address inequities in pain and pain care. Giacaman et al. synthesize the lived experiences of Palestinians in the West Bank with cancer pain, culminating with a powerful call to decolonize knowledge production. As part of the Oklahoma Study of Native American Pain Risk, Jones et al. explore cognitive-affective amplifiers of pain within a social context, focusing on relationships with interpersonal discrimination and cultural identification. Kissi et al. provide early evidence of racialized inequities in experimental pain and racialized discrimination in pain care among Black and Brown Belgian youth compared to White peers. Cassidy et al. address linguistic equity in pain assessment, validating a Modern Standard Arabic version of the Pain Disability Index. Together, these contributions highlight pain inequities in the global health context and underscore the role of historical and colonial structures, life course perspectives, and methodological approaches that shape how pain is experienced, assessed, and treated.

## Historical, cultural, and colonial structures

Pain inequities are embedded within historical systems of privilege and oppression that continue to shape lived experience, research, and care (1, 2). Engaging in historical and cultural study and reflection should inform how methodologies are constructed, data are analyzed, and results are disseminated (3–5). This entails an active process that contextualizes how pain researchers and their work are situated within higher-level structures and systems across history, allowing inequities across institutions, communities, and policies to be more clearly understood and dismantled (3–8).

Works from this Research Topic illustrate how pain is shaped by historical and structural forces. In their discussion, Kissi et al. acknowledge potential physiological mechanisms for pain inequities while emphasizing how individual experiences link to colonial legacies and racialized beliefs in the Belgian context. Jones et al. situate everyday interpersonal discrimination and cognitive-affective risk factors within colonial systems among Native American adults, with cultural identification reflecting how historical loss and acculturative stress remain embedded in pain experience. In their critical work, Giacaman et al. resist generations of ongoing colonial violence and oppression by decolonizing knowledge production through integrating physical and mental health, documenting patients’ use of local remedies, and emphasizing social support and solidarity for Palestinian survival. They further challenge the colonial and racist misrepresentation of the Muslim concept of *tawwakul* as fatalism by providing interpretation aligned with local communities. These works offer important insights for pain researchers on essential processes to ground their work in historical and cultural context and use dissemination to actively challenge systems of oppression.

## Community life course perspectives

Pain inequities represent collective experiences unfolding across the life course, shaped by shared histories, environments, and structural conditions (6, 9). Beyond the individual, experiences of discrimination and marginalization affect families and communities, while collective resources such as cultural practices and social solidarity can shape how pain is understood and managed in the face of injustice (7, 10).

Giacaman et al. demonstrate that despite profound structural violence, Palestinians living with cancer pain draw on family, community, and collective solidarity as essential sources of support. These resources, embedded within values such as *Sumud*, highlight the importance of viewing pain within its social and political context rather than solely as an individual experience. This perspective underscores the need to move beyond individually-focused intervention models toward approaches that recognize community-level strengths and barriers.

A life course perspective reveals how pain inequities can emerge early and compound over time. Findings from Kissi et al. show racialized disparities in pain tolerance and racialized discrimination in pain care among Belgian youth, suggesting developmental pathways through which inequities may persist into adulthood. Similarly, Jones et al. highlight how historical loss, discrimination, and acculturative stress shape chronic pain risk among Native American individuals across generations. Collectively, these studies emphasize that pain inequities are produced by multiple interacting systems of influence across the life course and point to the importance of learning from communities about their context-specific challenges and solutions.

## Building context-specific tools

Addressing pain inequities requires measurement tools that are historically, culturally, and linguistically situated (11–13). Measures that are detached from the contexts in which pain is experienced risk excluding or misrepresenting marginalized populations (13). This raises critical questions about who pain assessment tools are designed for and whose experiences they capture.

Cassidy et al.‘s validation of a Modern Standard Arabic version of the Pain Disability Index illustrates the importance of aligning psychometric rigor with cultural and linguistic relevance. Such efforts help ensure that pain-related disability is assessed in ways that are meaningful across populations, rather than relying on instruments developed within narrow sociocultural contexts.

Qualitative research plays a complementary role by capturing dimensions of pain that standardized measures can overlook (12). Giacaman et al.‘s narrative approach reveals spiritual, collective, and political meanings of pain that are central to patients’ lived experiences but rarely reflected in patient-reported outcomes. Integrating qualitative and quantitative methods allows researchers to understand where and how pain inequities manifest and supports more inclusive, context-responsive pain science (12).

## Conclusion

The articles in this Research Topic reinforce that pain is never “just skin-deep”. Pain experiences, assessments, and treatments are shaped by historical and colonial legacies and systems of oppression and privilege that operate across individual, community, and societal levels. Whether through documenting colonial continuities in European pain inequities, highlighting Indigenous and Palestinian experiences shaped by historical loss and occupation, or developing linguistically accessible assessment tools, these contributions challenge pain researchers and clinicians to confront the broader systems in which pain is experienced and managed. Meaningfully addressing pain inequities will require more than incremental methodological adjustments. It demands contextualizing work within historical and current systems of oppression, committing to community-engaged and life course-informed research, and investing in tools and approaches that honor cultural meaning and lived experience. Importantly, foundational knowledge and solutions are constructed by communities most affected by pain inequities. By working alongside communities, pain science can move toward more just, inclusive, and effective research and care.
